# Acute Pericarditis and Cancer Risk: A Matched Cohort Study Using Linked UK Primary and Secondary Care Data

**DOI:** 10.1161/JAHA.118.009428

**Published:** 2018-08-15

**Authors:** Kirstine Kobberøe Søgaard, Henrik Toft Sørensen, Liam Smeeth, Krishnan Bhaskaran

**Affiliations:** ^1^ Non‐Communicable Diseases Epidemiology London School of Hygiene and Tropical Medicine London United Kingdom; ^2^ Department of Clinical Epidemiology Institute of Clinical Medicine Aarhus University Hospital Aarhus N Denmark

**Keywords:** alcohol, lifestyle, neoplasia, obesity, smoking, Epidemiology, Pericardial Disease, Prognosis

## Abstract

**Background:**

We aimed to examine whether acute pericarditis is an indicator of undetected cancer and identify patient‐level factors associated with high cancer risk among patients presenting with pericarditis.

**Methods and Results:**

A population‐based matched cohort study was conducted using primary care data from the UK Clinical Practice Research Datalink linked to Hospital Episode Statistics. Patients with acute pericarditis (n=6530) were matched to a comparison cohort (n=26 111) on age, sex, calendar time, and general practice. We estimated cumulative cancer incidences, and calculated hazard ratios using Cox regression. Effect modification by patients' characteristics and lifestyle factors was examined, and we fitted a parsimonious model to evaluate absolute excess risk of later cancer among pericarditis patients by key patient‐level factors. We identified 728 and 1379 incidents of cancer among pericarditis patients and the comparison cohort (median follow‐up, 2.8 and 3.5 years). Pericarditis was associated with an elevated subsequent risk of any cancer (hazard ratio=3.03; 95% confidence interval, 2.74–3.36). The association was particularly pronounced 0 to 3 months after pericarditis (hazard ratio=23.56; 95% confidence interval, 18.00–30.83), but a more‐modest association remained thereafter (hazard ratio=1.95; 95% confidence interval, 1.48–2.57 after 3–12 months, and hazard ratio=1.40; 95% confidence interval, 1.21–1.62 after >12 month). Older individuals hospitalized with pericarditis and with combinations of obesity and smoking were at the highest excess risk of having a cancer diagnosis 3 to 12 months later, reaching 4.8%.

**Conclusions:**

Occult cancers may be going undiagnosed during the acute episode of pericarditis. Patients presenting with pericarditis and combinations of older age, obesity, smoking, and a need for hospitalization might warrant targeted investigations for cancer.

**Clinical Perspective**

**What Is New?**

- Whereas a diagnosis of underlying cancer is not uncommon during an episode of acute pericarditis, our study demonstrates that there is a high risk of cancer diagnosis for a prolonged period (notably 3–12 months and beyond) following pericarditis, suggesting that occult cancers may be going undiagnosed during the acute episode.
- Patients presenting with pericarditis and combinations of older age, obesity, current smoking, and a need for hospitalization had the highest absolute excess risks of later cancer and might warrant targeted investigations for cancer.
- The largest excess cancer incidences in the period 3 to 12 months following pericarditis were for hematological, colorectal, ovarian, kidney, pancreas, breast, and bladder cancers.

**What Are the Clinical Implications?**

- Increasing the awareness that some cancers may be being missed at the time of pericarditis, and about the patients and cancer types most affected, may help guide diagnostic workup and lead on to earlier cancer diagnoses.

## Introduction

Pericarditis is the most common disease of the pericardium and involves inflammation of the pericardium with or without pericardial effusion (pericardial exudate). Clinical presentation is characterized by chest pain, pericardial friction rub, and often with specific ECG changes. Acute pericarditis mainly affects the younger population, and it is usually benign and self‐limiting. However, pericarditis with effusion is a known complication to lung and breast cancer and has been reported also in patients with hematological malignancies, primary cardiac tumors, gastrointestinal cancer, and urogenital cancer.^1^, ^2^ The connection may stem from direct extension of cancer cells from nearby structures or hematogenous spread of abnormal cancer cells through the bloodstream.^3^ The link between pericarditis and cancer raises the question of whether pericarditis may be a useful marker of occult cancers and of importance in cancer detection.

A recent Danish population‐based cohort study by members of our group showed a high rate of several cancers, including lung, gastrointestinal tract, and urinary tract cancers, in addition to lymphoma and leukemia during the first 3 months after the pericarditis diagnosis.^4^ Of note, an increased risk was also observed for lung cancer, non‐Hodgkin's lymphoma, and bladder cancer diagnoses up to several years after the pericarditis diagnosis. Importantly, the increased risk of cancer was not confined to patients with pericardial effusion. These results suggest that a more‐thorough investigation may be warranted in some patients presenting with acute pericarditis to exclude these cancers, but confirmation of the observed associations is needed in independent data sets before such action might be recommended, and there is a need for data on which patients groups are most likely to benefit from additional investigations.

We therefore investigated the association between pericarditis and subsequent risk cancer in the United Kingdom. We also examined whether associations between pericarditis and cancer were modified by patient‐level factors, and evaluated absolute excess cancer risk among patients presenting with pericarditis, according to key patient‐level factors.

## Methods

Data, analytical methods, and study materials will not be made available to other researchers for purposes of reproducing the results or replicating the procedure. This is because we used secondary data under license and are not permitted to share these data further.

### Study Design and Data Sources

We carried out a population‐based matched cohort study using UK data from the Clinical Practice Research Datalink (CPRD^5^) and Hospital Episodes Statistics (HES^6^) databases. The CPRD is a collection of anonymized longitudinal patient‐level electronic health records from primary care in the United Kingdom. Data collection was initiated in 1987 and includes information on diagnoses, prescriptions, and various lifestyle characteristics as recorded by general practitioners (eg, smoking status, alcohol use, weight, and height).^5^ In the United Kingdom, more than 98% of the population is registered with a primary care general practitioner, and the National Health Service provides for general practitioner access which is free at the point of use. The CPRD includes data from more than 600 general practices totaling more than 14 million patients; it is broadly representative of the UK population in terms of age, sex, and ethnicity.^5^ Whereas the diagnoses in CPRD generally have a high validity,^7^ the validity of pericarditis specifically has not yet been examined. Therefore, to improve the ascertainment of pericarditis, in addition to cancers and comorbidities, we obtained individually linked HES data, which contain diagnostic information from in‐patient hospital care.^6^

### Study Population

We identified all people in the CPRD or HES with a first diagnosis of acute pericarditis between April 1, 1998 and December 31, 2015, with date of first diagnosis of pericarditis being the index date. Patients were identified using Read codes and *International Classification of Diseases, Tenth Revision* (*ICD‐10*) codes indicating acute pericarditis (covering idiopathic pericarditis, acute infectious pericarditis, pericarditis based on underlying systemic disease [eg, systemic lupus erythematosus, rheumatoid arthritis, or uremia], and pericardial effusion; Table S1). We identified a comparison cohort who had no history of pericarditis preceding the index date, matched 4:1 to the pericarditis patients by practice, sex, and age (within 3 years) and under follow‐up in the CPRD on the index date of the matched pericarditis patient. We excluded all people with a cancer diagnosis before the index date from both groups.

### Cancer Outcome

We identified all cancers through medical codes from the CPRD medical dictionary related to cancer (mapped to *ICD‐10* chapter 2 headings in a previous study^8^), and through *ICD‐10* codes in the HES, and restricted to first occurrence of cancer. We estimated risk for any cancer combined and for 14 individual cancers; the site‐specific cancer outcomes chosen for analysis included the most common cancers and was further informed by findings from previously published data on pericarditis and cancer risk.^4^, ^9^, ^10^

### Covariates

We classified patients based on underlying lifestyle‐related factors, namely tobacco smoking, alcohol use, and obesity. We used body mass index (BMI) calculated directly from weight and height records (weight/height^2^) and created 4 categories: underweight (BMI <18.5), normal weight (BMI=18.5–24.9), overweight (BMI=25.0–29.9), and obesity (BMI >30), using World Health Organization (WHO) grouping of overweight and obesity. Details on processing, cleaning, and representativeness of CPRD BMI data have been previously described.^8^, ^11^ We categorized smoking status into never‐smoker, current smoker, and ex‐smoker, and similarly alcohol use into never‐use, current use, and ex‐use. We excluded patients with missing data in the lifestyle‐related covariates; to identify any possible selection issues arising from this approach, we descriptively compared those with complete versus missing data in terms of other covariates (Table S2).

For descriptive purposes, we obtained information on acute myocardial infarction registered close to the index date (between 30 days before and 7 days after) and retrieved information on connective tissue disease diagnoses for patients back to 1987 or start of current up‐to‐standard follow‐up (whichever came later).

### Statistical Analyses

#### Absolute and relative risks of cancer associated with a pericarditis diagnosis

We followed all patients from the diagnosis of pericarditis (or index date for matched comparison patients) until the first occurrence of cancer (ie, the outcome), date of death, transfer out of the CPRD, or December 31, 2015, whichever came first. We used Kaplan–Meier techniques to compute cumulative incidence of cancer (absolute risks) in the exposed and unexposed groups. We used Cox regression to compare cancer risks in patients with pericarditis and controls; we stratified by matched set to account for the matching by practice, sex, age, and calendar date. We did not adjust for further covariates, such as smoking, because our aim was to assess the extent to which pericarditis predicted future cancer risk: we did not hypothesize that pericarditis would *cause* later cancer. We computed hazard ratios (HRs) for all cancers combined and separately, classifying follow‐up periods into 0 to 3 months (including day 90), 3 to 12 months (including day 365), and >12 months following the pericarditis diagnosis/index date. We also computed HRs according to whether or not the pericarditis patient was recorded as being hospitalized with this condition and whether or not the patient had pericardial effusion.

We then investigated modification of the association between pericarditis and any cancer by sex, age, and lifestyle factors (categorized as described above) by fitting interaction terms and carrying out likelihood ratio tests. The stratum‐specific HR estimates were obtained by multiplying main effect and interaction terms. We also computed HRs according to whether or not the pericarditis patient was recorded as being hospitalized with this condition and whether or not the patient had pericardial effusion.

#### Absolute excess cancer risk after pericarditis by patient‐level factors

Finally, we developed a parsimonious model for absolute excess cancer risk among pericarditis patients, restricting to the risk period between 3 and 12 months after pericarditis diagnosis. The first 3 months were excluded because many cancer diagnoses in the period immediately after pericarditis may have been incidental findings from routine clinical investigations into the pericarditis (eg, by chest x‐ray, blood counts) or be occult cancers successfully identified during the acute episode. In contrast, cancers diagnosed in the 3‐ to 12‐month period were likely diagnosed in a separate episode of care from the pericarditis and could be a target for earlier detection at the time of the pericarditis. To identify patient‐level factors associated with absolute excess cancer risk after pericarditis, a 2‐stage modeling process was carried out based on the methods of relative survival modeling.^12^ First, we fitted a Poisson model for incident cancer in the control group and generated predictions from this model to obtain expected numbers of cancers in the absence of pericarditis, within strata defined by the covariates in the model (which were age at index, sex, smoking status, alcohol use, and BMI); numbers of excess cancers in the pericarditis group were then derived within covariate strata by subtracting these expected numbers from the observed numbers of incident cancers in each stratum; finally, a second Poisson model was developed restricted to the pericarditis group, with the numbers of excess cancers as the outcome, and beginning with the above covariates plus hospitalization and pericardial effusion. We used backward elimination (with *P*‐value threshold of 0.20 for removal) to reduce the model to a limited number of the most important factors associated with excess cancer risk. The final model from the backward elimination process was used to generate expected absolute excess cancer risks in the 3‐ to 12‐month period after pericarditis, for every combination of factors included in the final model.

#### Sensitivity analyses

In a sensitivity analysis, we excluded patients diagnosed with pericarditis and cancer on the same date, to make sure they could not explain the entire excess risk for the first follow‐up period.

Statistical analyses were done using the STATA statistical software package (release 14; StataCorp LP, College Station, TX). The study was approved by the Independent Scientific Advisory Committee for MHRA Database Research (approval number 16_230RA2), and the London School of Hygiene and Tropical Medicine ethics committee (approval number 11964). The need to obtain informed consent from patients has been waived for observational studies using pseudonymized data from the CPRD and HES.

## Results

### Characteristics

In total, we identified 6530 patients with incident pericarditis and a comparison cohort of 26 111 people without pericarditis. Pericarditis patients were identified in the CPRD (n=2371), HES (n=3776), or in both registries (n=383). Among the pericarditis patients, 2397 (37%) were recorded as acute unclassified, 355 (5%) acute infectious, 3734 (57%) with pericardial effusion, and 44 (<1%) uremic or postinfarction pericarditis, or with underlying autoimmune disease.

Patients and comparison cohort members were followed for a median of 2.8  (interquartile range, 0.8–5.9) and 3.5 years (interquartile range, 1.5–6.7), respectively. Table 1 describes the characteristics of the pericarditis patients and the matched comparison cohort. Ever smoking, recent myocardial infarction, and history of connective tissue disease were more common among pericarditis patients than controls.

**Table 1:** Descriptive of 6530 Patients With Acute Pericarditis and a Matched Comparison Cohort of 26 111 People Without Pericarditis

	Pericarditis, N (%)	Comparison Cohort, N (%)
Men^a^	3983 (61)	15 925 (61)
Age category, y^a^		
<50	2272 (35)	9088 (35)
50 to 69	2185 (33)	8740 (33)
>70	2073 (32)	8283 (32)
Calendar period^a^		
1998–2003	956 (15)	3821 (16)
2004–2008	1963 (30)	7852 (30)
2009–2011	1574 (24)	6295 (24)
2012–2015	2037 (31)	8143 (31)
Smoking		
Nonsmoker	2439 (37)	11 424 (44)
Current smoker	1437 (22)	5055 (19)
Ex‐smoker	2491 (38)	8397 (32)
Missing data	163 (3)	1235 (5)
Alcohol		
Nonuser	630 (10)	2235 (9)
Current user	4438 (68)	17 757 (68)
Ex‐user	658 (10)	2125 (8)
Missing data	804 (12)	3994 (15)
BMI categories		
Underweight (<18.5)	191 (3)	448 (2)
Normal weight (18.5–24.9)	2062 (32)	8247 (31)
Overweight (25.0–29.9)	2057 (32)	8327 (32)
Obese (>30)	1408 (22)	5128 (20)
Missing data	812 (12)	3961 (15)
Recent myocardial infarction^b^	319 (5)	15 (0.1)
Connective tissue disease^c^	412 (6)	751 (3)

BMI indicates body mass index.

a Matching factor.

b Within 60 days before or 7 days after pericarditis.

c Ever before or 7 days after.

### Cumulative Cancer Incidences in the Pericarditis and Control Groups

During complete follow‐up, 728 incident cancers were observed among pericarditis patients, with lung cancer as the most frequent type (n=175), followed by non‐Hodgkin's lymphoma (n=53), prostate cancer (n=51), colorectal cancer (n=50), breast cancer (n=43), and leukemia (n=39; Table 2). A total of 367 of 728 (50%) of cancers were diagnosed within the first 3 months after pericarditis (of these 247 on the same date as the pericarditis), 77 were diagnosed during the 3‐ to 12‐month follow‐up period, and 284 in 1 or more years following the episode with pericarditis. The cumulative absolute risk of any cancer among patients with pericarditis versus controls was 5.7% versus 0.3% at 3 months, 7.2% versus 1.2% at 12 months, and 12.2% versus 5.5% at 5 years (Table 2). Absolute risks in the pericarditis group were highest for lung cancer at 2.2% after 3 months, 2.3% at 12 months, and 2.8% at 5 years. Females, patients aged >70 years, and those with pericardial effusion had the highest 3‐month risk of cancer (Table S3).

**Table 2:** Total Number of Cancers (N) and Cumulative Incidences in Percentage With 95% CI, by Follow‐up Periods

		Pericarditis				Comparison Cohort		
	N	3 Months	12 Months	5 Years	N	3 Months	12 Months	5 Years
Any cancer	728^a^	5.7 (5.2–6.3)	7.2 (6.5–7.8)	12.2 (11.3–13.3)	1379	0.3 (0.2–0.4)	1.2 (1.1–1.4)	5.5 (5.1–5.9)
Oral	6	0.0 (‐)^b^	0.0 (0.0–0.1)	0.1 (0.0–0.3)	3	0.0 (0.0–0.0)	0.0 (0.0–0.0)	0.0 (0.0–0.0)
Heart/mediastinum/pleura	7	0.1 (0.0–0.2)	0.1 (0.1–0.2)	0.1 (0.1–0.2)	0	0.0 (…)^b^	0.0 (…)^b^	0.0 (…)^b^
Lung	175	2.2 (1.9–2.6)	2.3 (2.0–2.7)	2.8 (2.4–3.3)	140	0.0 (0.0–0.0)	0.1 (0.1–0.2)	0.6 (0.5–0.7)
Breast	43	0.4 (0.2–0.8)	0.7 (0.4–1.2)	2.4 (1.7–3.4)	115	0.1 (0.1–0.2)	0.3 (0.2–0.5)	1.3 (1.0–1.6)
Ovary	12	0.1 (0.1–0.2)	0.2 (0.1–0.3)	0.2 (0.1–0.3)	21	0.0 (0.0–0.0)	0.0 (0.0–0.0)	0.1 (0.1–0.2)
Colorectal	50	0.2 (0.1–0.3)	0.3 (0.2–0.5)	0.9 (0.6–1.2)	145	0.0 (0.0–0.1)	0.2 (0.1–0.2)	0.6 (0.5–0.8)
Pancreas	18	0.1 (0.0–0.2)	0.1 (0.1–0.3)	0.4 (0.2–0.6)	32	0.0 (0.0–0.0)	0.0 (0.0–0.1)	0.2 (0.1–0.2)
Kidney	11	0.1 (0.0–0.2)	0.1 (0.1–0.3)	0.2 (0.1–3.3)	16	0.0 (0.0–0.0)	0.0 (0.0–0.0)	0.1 (0.0–0.1)
Bladder	25	0.1 (0.0–0.2)	0.1 (0.1–0.3)	0.5 (0.3–0.8)	71	0.0 (0.0–0.1)	0.1 (0.1–0.1)	0.3 (0.2–0.4)
Prostate	51	0.1 (0.0–0.2)	0.2 (0.1–0.4)	1.0 (0.7–1.4)	228	0.0 (0.0–0.1)	0.2 (0.1–0.2)	0.9 (0.8–1.1)
Brain/CNS	9	0.0 (0.0–0.1)	0.1 (0.0–0.2)	0.2 (0.1–0.4)	16	0.0 (0.0–0.0)	0.0 (0.0–0.0)	0.0 (0.0–0.1)
Non‐Hodgkin's lymphoma	53	0.6 (0.4–0.8)	0.7 (0.5–0.9)	1.0 (0.8–1.4)	44	0.0 (…)^b^	0.0 (0.0–0.1)	0.2 (0.1–0.3)
Hodgkin's lymphoma	8	0.0 (0.1–0.2)	0.1 (0.1–0.2)	0.2 (0.1–0.3)	1	0.0 (…)^b^	0.0 (…)^b^	0.0 (0.0–0.0)
Leukemia	39	0.3 (0.2–0.5)	0.4 (0.3–0.6)	0.7 (0.5–1.0)	39	0.0 (0.0–0.0)	0.0 (0.0–0.1)	0.2 (0.1–0.2)

CI indicates confidence interval; CNS, central nervous system.

a A total of 728 cancers were diagnosed among pericarditis patients: 0 to 3 months, n=357 (247 on the same date as the pericarditis); 3 to 12 months, n=87; and >12 months, n=284.

b 95% CI not calculated because of 0 events.

### Hazard Ratios for Association Between Pericarditis and Cancer

Patients with pericarditis had a higher risk of any cancer during complete follow‐up (HR=3.03; 95% confidence interval [CI], 2.74–3.36) compared with their comparators from the general population. All individual cancers, except for prostate, were associated with an increased overall cancer risk, ranging from ≈2‐fold increases in risk for breast, colorectal, and bladder cancer, to HRs between 6.4 and 7.6 for non‐Hodgkin's lymphoma, leukemia, and lung cancer (Table 3).

**Table 3:** Hazard Ratios^a^ (HR) of Cancer Among 6530 Patients With Pericarditis and a Matched Comparison Cohort of 26 111 People Without Pericarditis

	No. of Cancer Among Pericarditis Patients/Controls, HRs^a^ (95% CI)							
	n	Complete Follow‐up	n	0 to 3 Months	n	3 to 12 Months	N	>12 Months
Any cancer	728/1379	3.0 (2.7–3.4)	367/77	23.6 (18.0–30.8)	77/213	2.0 (1.5–2.6)	284/1089	1.4 (1.2–1.6)
Oral	6/3	7.0 (1.7–28.3)	0/1	b	1/1	b	5/1	b
Heart/mediastinum/pleura	7/0	>27.1^c^	6/0	b	1/0	b	0/0	b
Lung	175/140	7.6 (5.9–9.9)	143/4	190 (60.8–598)	5/25	1.1 (0.4–2.9)	27/111	1.2 (0.8–2.0)
Breast	43/115	2.2 (1.5–3.3)	10/10	5.00 (2.0–12.7)	6/21	1.7 (0.6–4.4)	27/81	1.9 (1.2–3.1)
Ovary	12/21	3.5 (1.6–8.0)	8/1	32.0 (4.0–255)	3/1	11.2 (1.2–108)	1/19	0.3 (0.0–2.2)
Colorectal	50/145	1.7 (1.2–2.4)	11/11	4.8 (2.0–11.5)	8/24	2.0 (0.8–4.6)	31/110	1.3 (0.9–2.1)
Pancreas	18/32	3.2 (1.6–3.4)	5/1	20.0 (2.4–171)	3/7	3.0 (0.7–13.4)	10/24	2.0 (0.8–5.0)
Kidney	11/16	3.3 (1.5–7.5)	5/2	10.0 (1.4–51.4)	2/1	8.0 (0.7–88.2)	4/13	1.5 (0.5–4.8)
Bladder	25/71	2.2 (1.3–3.7)	3/10	1.3 (0.4–4.3)	4/13	2.2 (0.7–7.6)	18/48	2.5 (1.3–4.7)
Prostate	51/228	1.2 (0.8–1.6)	6/8	4.3 (1.3–14.4)	7/31	1.0 (0.4–2.3)	38/189	1.1 (0.7–1.6)
Brain/CNS	9/16	2.4 (1.0–5.9)	2/1	b	1/1	b	6/14	b
Non‐Hodgkin's lymphoma	53/44	6.4 (4.1–10.1)	37/0	>148^c^	5/8	2.3 (0.8–7.2)	11/36	1.6 (0.8–3.3)
Hodgkin's lymphoma	8/1	31.1 (3.9–248)	7/0	b	0/0	b	1/1	b
Leukemia	39/39	7.0 (4.0–12.4)	20/2	80.0 (10.7–596)	5/4	6.7 (1.6–27.9)	14/33	2.4 (1.1–5.2)

Person‐time at risk: pericarditis patients: 25 593, comparison cohort: 117 655. CI indicates confidence interval; CNS, central nervous system.

a Adjusting for matching factors by design (age [±3 years], sex, and practice).

b Not estimated because of limited number of events.

c There were 0 events in the comparison cohort; therefore, we added 1 event to the comparison cohort to give a lower bound for HR.

Associations between pericarditis and cancer varied by time since pericarditis, with an HR of 23.56 (95% CI, 18.00–30.83) for any cancer in the first 3 months, reducing to HR=1.95 (95% CI, 1.48–2.57) between 3 and 12 months, and HR=1.40 (95% CI, 1.21–1.62) 1 or more years after the pericarditis. Positive associations between pericarditis and individual cancers were also noted within the first 3 months after the index date for all cancer sites investigated except bladder cancer, though CIs for the magnitude of the HRs were wide for some outcomes. HRs dropped markedly within the 3‐ to 12‐month period for most cancers, though there remained a 7‐fold elevated risk for leukemia; for several other outcomes, HR point estimates did remain elevated, but CIs were too wide to be conclusive. One or more years after the index date, there was evidence of a sustained increased risk of breast cancer: HR=1.82 (95% CI, 1.17–3.14); leukemia, HR=2.35 (95% CI, 1.07–5.15); and bladder cancer, HR=2.51 (95% CI, 1.33–4.71).

The HR associated with hospitalized pericarditis was higher than for pericarditis handled in the primary care setting only (HR for any cancer=3.38 [95% CI, 3.00–3.80] versus HR=2.26 [95% CI, 1.86–2.76], respectively). We also found that pericardial effusion was associated with a higher overall HR than pericarditis without effusion (HR=3.59 [95% CI, 3.18–4.06] versus HR=2.04 [95% CI, 1.69–2.46]).

### Effect Modification by Individual‐Level Factors

We found that several characteristics clearly modified cancer risk (Figure). The association between pericarditis and cancer risk was larger at younger ages (HR=6.62 [95% CI, 4.76–9.22] at age <50, and 2.66 [95% CI, 2.29–3.08 at age ≥70 years]) and larger among current smokers (HR=4.22 [95% CI, 3.25–5.50], compared with 2.65 [95% CI, 2.19–3.21] among nonsmokers). There was some evidence for a larger association with cancer in women than in men, but the direction of potential interaction by alcohol use and BMI categories was less clear (Figure).

**Figure 1:**
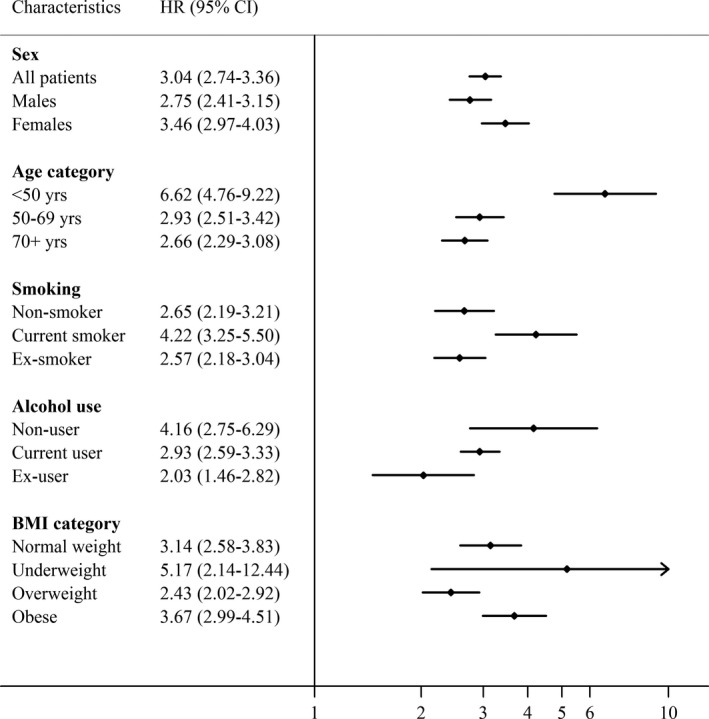
HRs stratified by characteristic and lifestyle factors. HRs adjusted for matching factors by design (age, sex, practice, and calendar period). Likelihood ratio test for interaction: sex, *P*=0.03; age category, *P*<0.001; smoking status, *P*=0.006; alcohol use, *P*=0.03; body mass index, *P*=0.02. BMI indicates body mass index; CI, confidence interval; HR, hazard ratio.

### Absolute Excess Risk of Cancer Diagnosis 3 to 12 Months After Pericarditis

Among pericarditis patients, 77 cancers were diagnosed in the 3‐ to 12‐month period after diagnosis (Table 3). The largest absolute excess incidences (per 1000 person‐years) in this period (incidence in pericarditis patients minus incidence in controls) were for leukemia (0.81), non‐Hodgkin's lymphoma (0.64), and colorectal (0.55), ovarian (0.54), kidney (0.35), pancreas (0.29), breast (0.29), and bladder cancers (0.23). A backward elimination model selection process to develop a model for the absolute excess risk of cancer in the 3‐ to 12‐month period after pericarditis led to a final model, including age, BMI (classified as obese versus nonobese only, because the overweight category was not predictive in the final model), current smoking, and hospitalization at the time of pericarditis. Expected excess risks of cancer by 12 months from this model are shown in Table 4 and ranged from 0.3% (95% CI, 0.1–0.7) in the lowest‐risk subgroup (younger nonobese nonsmoking individuals diagnosed without hospitalization) to 4.8% (95% CI, 2.2–10.8) in the highest‐risk subgroup (older obese current smokers hospitalized for pericarditis).

**Table 4:** Absolute Excess Risk of Cancer in the 3‐ to 12‐Month Period After Pericarditis, by Key Covariates

	Excess risk in % (95% CI)		
	Age <50 y	Age 50 to 69 y	Age >70 y
Not hospitalized			
Not obese, nonsmoker	0.3 (0.1–0.7)	0.8 (0.4–1.6)	1.2 (0.6–2.4)
Not obese, smoker	0.5 (0.2–1.1)	1.3 (0.6–2.9)	2.0 (0.9–4.7)
Obese, nonsmoker	0.5 (0.2–1.2)	1.3 (0.7–2.7)	2.0 (0.9–4.4)
Obese, smoker	0.7 (0.3–2.0)	2.2 (0.9–5.1)	3.3 (1.3–8.6)
Hospitalized			
Not obese, nonsmoker	0.4 (0.2–1.0)	1.2 (0.7–2.1)	1.8 (1.2–2.8)
Not obese, smoker	0.7 (0.3–1.6)	2.0 (1.0–3.9)	3.0 (1.5–5.9)
Obese, nonsmoker	0.7 (0.3–1.7)	2.0 (1.1–3.5)	3.0 (1.7–5.2)
Obese, smoker	1.1 (0.4–2.8)	3.2 (1.6–6.7)	4.8 (2.2–10.8)

Analysis restricted to people cancer free at 3 months after pericarditis. CI indicates confidence interval.

### Sensitivity Analysis

A total of 357 patients were diagnosed with cancer within the first 3 months; among these, 307 were diagnosed during first months, and of these, 247 were registered on the same date as pericarditis. We repeated the main analyses after exclusion of the 247 patients diagnosed with cancer at the same date. The HR for overall cancer during complete follow‐up was 1.92 (95% CI, 1.71–2.15), and for the first 3 months, HR was 7.70 (95% CI, 5.65–10.49); though attenuated, the results were robust and the overall conclusion unchanged.

## Discussion

### Key Findings

In this study, we found that pericarditis may be a marker of occult cancer. Patients with pericarditis were at a 3‐fold increased risk of a cancer diagnosis compared with people from the matched comparison cohort. The most marked increase in risk was in the first 3 months after pericarditis, likely reflecting cancers diagnosed during the episode of care in which the pericarditis was recorded. However, even after the first 3 months, we found a substantially elevated risk of cancer in the pericarditis group, indicating that the pericarditis diagnoses may, in some cases, have been a missed opportunity for earlier cancer diagnosis during the acute episode. In relative terms, pericarditis was associated with a particularly elevated risk of cancer in people aged <50 years and in current smokers, but our model for absolute excess cancer risks suggested that the greatest opportunity for early cancer detection may be among older people, obese people, current smokers, and those hospitalized at the time of pericarditis.

### Comparison With Previous Studies

A recent Danish population‐based cohort study including 13 759 patients with acute pericarditis (hospital‐based diagnosis) examined the risk of subsequent cancer in patients with no previous cancer history.^4^ In our present study using data from the CPRD and HES, we confirmed the key findings from the study on the Danish population in a different setting, including the highly elevated risk of a cancer diagnosis within the first 3 months, decreasing thereafter. Importantly, we have also built on these findings and added to their clinical relevance by identifying patient groups who may be at highest risk of missed cancers and warrant targeted investigations at the time of pericarditis. Some important differences between the 2 studies should be noted. The Danish cohort were younger than our cohort (median age was 49 versus 60 years) and had a longer follow‐up (median of 6.4  versus 2.8 years). The prevalence of recent myocardial infarction and connective tissue disease were comparable. In the Danish cohort, overall relative risk of cancer associated with pericarditis was 1.5, lower than ours of 3.0. Similarly, in the first 3 months, relative risk estimates were smaller in the Danish study (12‐ versus 24‐fold increase). In regard to cancer‐specific relative risks, there was agreement in direction and size for colon, bladder, and prostate, whereas the estimates were substantially higher for oral, lung, breast, pancreatic, kidney, and ovary, as well as lymphoma and leukemia in the UK cohort. In both cohorts, relative risks in the follow‐up period beyond 1 year remained clearly elevated for oral cancer and bladder cancer, and results were in agreement also for lung, colon, ovary, kidney, and prostate cancer as well as non‐Hodgkin's lymphoma, though for several cancers, including lung cancer, the present study did not have sufficient power to rule out that the modest observed association was attributable to chance given that the CI included unity. By contrast, in the Danish cohort, there was no excess occurrence of breast cancer and leukemia beyond 1 year of follow‐up, whereas the HR of these cancers remained >2‐fold elevated in the UK cohort. Consistent with the Danish study, we confirmed that the increased risk of cancer was not confined to pericarditis patients with a record of pericardial effusion, and that the relative risk of cancer was higher in women than men. Differences between the 2 studies may be explained by a number of things. Differences in distribution of patient characteristics (eg, age and smoking) in addition to length of follow‐up may have impacted on the results. Also, for some cancers, incidence rates may be different in the United Kingdom and Denmark. This would impact on both the cancer‐specific as well as the overall HR. Finally, there could be differences in recording practices between the United Kingdom and Denmark.

### Strengths and Limitations

The diagnosis of acute pericarditis is based on 2 of the following criteria: (1) chest pain, (2) pericardial friction‐rub, (3) characteristic ECG changes, and (4) pericardial effusion on 2‐dimensional echocardiography. Elevated inflammatory markers and evidence of inflammation by imaging modalities may support diagnosis. The diagnosis can be challenging given that the only reliable criterion may be symptomatic chest pain, whereas criteria 2 to 4 can be difficult to confirm with the standard diagnostic workup in the general practitioner setting. In the hospital setting, cardiac magnetic resonance imaging has a high sensitivity for detecting even smaller pericardial effusions. Our cohort was based on data from the CPRD and HES, which are considered to have a good generalizability both to the UK population and to comparable countries. Pericarditis diagnoses, as recorded in the CPRD and HES, have not been specifically validated, so there may have been some misclassification in our exposure measure, but our combined use of primary care and hospital data should have improved sensitivity and allowed us to include patients who may have had pericarditis without hospital admission.

More broadly, CPRD diagnosis data have been shown to have good validity, including for cancer diagnoses, which have a high concordance with data from other sources.^7^ The large size of the CPRD gave us power to investigate site‐specific cancers and stratify by time since pericarditis. Even in this large database, though, some results for site‐specific cancers had limited precision because of few events, particularly when we also stratified by time since pericarditis.

We aimed to examine whether pericarditis was a marker of prevalent undiagnosed cancer and therefore excluded all patients known to have cancer at the time of pericarditis diagnosis. Still, for some patients, admission for pericarditis may have prompted more‐thorough workup leading to cancer diagnosis, most evidently influencing the first follow‐up period (3 months). In particular, patients were likely examined with a chest x‐ray or other imaging, which could have revealed a previously unrecognized lung cancer or lymphoma, even if the pericarditis itself was not related to the cancer. Relative risk of cancer may thus be inflated during the first follow‐up period. Nevertheless, the finding of an increased risk for a number of cancer sites more than 3 months, and sometimes more than 12 months, after the acute pericarditis episode suggests that this incidental identification of cancers during pericarditis workup cannot explain all of the observed associations. In developing our final model for absolute excess cancer risk among the pericarditis group, we dealt with this issue by excluding the first 3 months of follow‐up to reduce the influence of cancers that may have been incidental findings unrelated to the pericarditis, and for which there would in any case have been little opportunity for earlier detection.

### Clinical and Public Health Implications

The majority of patients with pericarditis will have a self‐limiting disease and will mainly only undergo workup aiming to exclude myocardial infarction (ie, ECG, echocardiography, biomarkers for acute cardiac ischemia, or chest x‐ray).^9^ According to guidelines, patients diagnosed in more‐recent years also should have assessment of markers of inflammation (ie, C‐reactive protein and/or erythrocyte sedimentation rate), white blood cell count with differential count, renal function, and liver tests).^9^ Additional testing with imaging examination is indicated in high‐risk patients defined by the presence of clinical indicators (eg, large pericardial effusion). It is clinically important to know whether patients presenting with first‐time pericarditis should be investigated more thoroughly to exclude particular cancers. The National Institute for Health and Care Excellence (NICE) guideline proposed a 3% positive predictive value threshold for markers of cancer, meaning that in cases where specific markers are associated with a >3% risk of cancer, the patient should follow the recommendations for “suspected cancer pathway referrals.”^13^ In our cohort, several subgroups of older people with combinations of obesity, current smoking, and hospitalization for pericarditis had absolute excess cancer risks of >3% in the 3‐ to 12‐month period following pericarditis and thus may warrant referral and investigations to specifically exclude cancer. The largest excess incidences in this period were for hematological malignancies (in particular, non‐Hodgkin's lymphoma and leukemia) and colorectal, ovarian, kidney, pancreas, breast, and bladder cancers; hence, these would appear the most important cancers to exclude following a diagnosis of pericarditis. This would clearly result in economic and clinical costs, but these may be more than offset by the gains of earlier detection^14^; a formal study of cost‐benefit in this scenario may be informative.

### Conclusion

We confirmed that pericarditis may signal an undetected cancer. Although substantial numbers of cancers are diagnosed during the acute pericarditis episode, our findings of prolonged increased risks of some types of cancer strongly suggest that many occult cancers may be going undiagnosed during the acute episode. Older people, obese people, current smokers, and those hospitalized at the time of pericarditis were at particularly high absolute excess risks of later cancers, and the presence of combinations of these risk factors in a patient presenting with pericarditis may indicate a need for referral or investigations to exclude cancer.

## Sources of Funding

Bhaskaran is funded by a Wellcome Trust/Royal Society Sir Henry Dale fellowship (107731/Z/15/Z). The sponsors had no role in the design, analysis, or writing up of this study.

## Disclosures

None.

## Supporting information


**Table S1.** Codes Indicating Acute Pericarditis in the Clinical Practice Research Datalink and in the Hospital Episodes Statistics
**Table S2.** Descriptive for 6337 Patients With and 26 304 Patients Without Missing Data
**Table S3.** Cumulative Incidence of Any Cancer at 3 Months, by Characteristics
