# Supplementary material for: Acute Pericarditis and Cancer Risk: A Matched Cohort Study Using Linked UK Primary and Secondary Care Data
Source: J Am Heart Assoc. 2018 Aug 15;7(16):e009428. doi: 10.1161/JAHA.118.009428 (PMC6201410; doi:10.1161/JAHA.118.009428)
Supplement: Supplementary file 1 — Table S1. Codes Indicating Acute Pericarditis in the Clinical Practice Research Datalink and in the Hospital Episodes Statistics Table S2. Descriptive for 6337 Patients With and 26 304 Patients Without Missing Data Table S3. Cumulative Incidence of Any Cancer at 3 Months, by Characteristics [file JAH3-7-e009428-s001.pdf]

SUPPLEMENTAL MATERIAL

Table S1. Codes indicating acute pericarditis in the Clinical Practice Research Datalink and in the Hospital Episodes Statistics.

Clinical Practice Research Datalink		Hospital Episodes Statistics
**Medcode**	**Readterm**	**ICD-10 code**
	**Acute pericarditis, idiopathic**	I30.0; I30.8; I30.9
3399	Acute pericarditis	
14646	Other and unspecified acute pericarditis	
27606	Acute pericarditis – unspecified	
100907	[X]Other forms of acute pericarditis	
36755	Acute pericarditis NOS	
15089	Acute pericarditis in diseases EC NOS	
29551	Acute pericarditis in diseases EC	
108258	[X]Pericarditis in other diseases classified elsewhere	
5690	[X]Pericarditis in other diseases classified elsewhere	
	**Acute infectious pericarditis**	I30.1; I32.0; I32.1
8411	Viral pericarditis NOS	
9113	Coxsackie pericarditis	
16803	Meningococcal pericarditis	
16996	TB - acute pericarditis	
36496	Acute pericarditis – pneumococcal	
57126	Acute pericarditis – tuberculous	
59102	Acute idiopathic pericarditis	
64481	Acute purulent pericarditis unspecified	
65897	Syphilitic pericarditis	
104081	Acute pericarditis – staphylococcal	
	**Pericardial effusion**	I31.3
2520	Pericardial effusion – acute	
18293	Pericardial effusion – noninflammatory	
45311	Pericardial effusion – acute	
105192	Pericardial effusion	
	**Pericarditis, disease elsewhere classified**	I32.8
11920	Systemic lupus erythematosus with pericarditis	
40956	Acute pericarditis – uraemic	
35119	Post infarction pericarditis	
	Rheumatoid arthritis	

Table S2. Descriptive for 6,337 patients with and 26,304 patients without missing data.

	With missing data	Without missing data
**Men**	4533 (72)	15,375 (58)
**Median age, years (IQR)**	39 (22-63)	63 (48-75)
**Median follow-up, years (IQR)**	2.9 (1.2-5.9)	3.5 (1.4-6.7)
**Recent myocardial infarction***	128 (2.0)	1035 (3.9)
**Connective tissue disease†**	133 (1.7)	884 (3.1)

* Within 60 days before or 7 days after pericarditis

† Ever before or 7 days after

Table S3. Cumulative incidence of any cancer at 3 months, by characteristics.

	Cumulative incidence % (95% CI)	
	**Pericarditis patients**	**Comparison cohort**
**All patients**	5.7 (5.2-6.3)	0.3 (0.2-0.4)
**Pericardial effusion**	9.1 (8.2-10.1)	NA
**Men**	4.5 (3.9-5.2)	2.4 (1.8-3.4)
**Women**	7.6 (6.6-8.7)	3.9 (2.9-5.3)
**Age <50 years**	2.7 (2.1-3.5)	0.0 (0.0-0.1)
**Age 50-69 years**	6.8 (5.9-8.0)	0.2 (0.2-0.4)
**Age >70 years**	7.8 (6.8-9.1)	0.7 (0.5-0.9)
**Recent myocardial infarction***	0.7 (0.2-2.6)	NA
**Connective tissue disease†**	4.9 (3.1-7.5)	1.0 (0.5-2.0)
**Current smoker**	7.4 (6.2-8.9)	0.2 (0.1-0.4)
**Current user of alcohol**	5.5 (4.8-6.2)	0.3 (0.2-0.4)
**Underweight (<18.5)**	8.6 (5.3-13.6)	0.5 (0.1-1.8)
**Normal weight (18.5-24.9)**	6.2 (5.2-7.3)	0.3 (0.2-0.4)
**Overweight (25-29.9)**	5.2 (4.3-6.3)	0.3 (0.2-0.5)
**Obese (>30)**	5.5 (3.6-5.8)	0.4 (0.3-0.6)

* Within 60 days before or 7 days after pericarditis. † Ever before or 7 days after
NA, not applicable
